# Effects of Pre-ovulatory Follicular Fluid of Repeat Breeder Dairy Cows on Bovine Fertility Transcriptomic Markers and Oocytes Maturation and Fertilization Capacity

**DOI:** 10.3389/fvets.2021.670121

**Published:** 2021-04-23

**Authors:** Mojtaba Kafi, Mehran Ghaemi, Mehdi Azari, Abdolah Mirzaei, Samad Azarkaman, Yusof Torfi

**Affiliations:** ^1^Department of Clinical Sciences, School of Veterinary Medicine, Shiraz University, Shiraz, Iran; ^2^Department of Pathobiology, School of Veterinary Medicine, Shiraz University, Shiraz, Iran

**Keywords:** endometritis, follicular fluid, gene expression, lipopolysaccharide, repeat breeder cow

## Abstract

The current study aimed to determine the effects of the preovulatory follicular fluid (FF) of normal heifer (NH) and repeat breeder cows with subclinical endometritis (SCE) or without (nSCE) on oocyte maturation (Experiment 1) and fertilization rates (Experiment 2). Moreover, the pattern of gene expression of cumulus oocyte-complexes was evaluated in Experiment 1. In Experiment 1, nuclear maturation in the nSCE group was higher, compared to that in the SCE group (*P* = 0.05). In addition, the oocyte nuclear maturation in the normal heifer was significantly higher, in comparison to that of SCE groups (*P* < 0.05). Furthermore, the mean percentage of normal oocyte fertilization was higher in the nSCE group, compared to that in the SCE group (*P* < 0.05). The expressions of growth differentiation factor, *GDF9;* steroidogenic acute regulatory, *StAR* and follicle-stimulating hormone receptor, *FSHr* in the NH group were significantly higher, compared to those in SCE and nSCE groups (*P* < 0.05). Moreover, the expressions of all genes in the nSCE group were not significant, in comparison to those in the SCE group (*P* > 0.05). The supplementation of oocyte maturation medium with FF from pre-ovulatory follicles of repeat breeder cows resulted in less oocyte maturation and cumulus cell expansion. In conclusion, the lower fertility in RB cows could be ascribed to the lower oocyte maturation rate and less expression of *GDF9, StAR*, and *FSHr* in the cumulus-oocyte complexes.

## Introduction

Repeat breeder (RB) cows are described as subfertile cows that do not become pregnant at least after three consecutive services. Different factors, including uterine infections, hormonal imbalances, mismanagement in the artificial insemination, nutritional factors, and genetic disorders may result in the occurrence of RB syndrome in dairy cows (1). A related study reported subclinical endometritis (SCE) in 52.7% of RB cows (2). In addition, researchers denoted that SCE is a major risk factor for reproductive failure resulting in lower reproductively of dairy cows (3, 4). On the contrary, it was found that SCE is not the cause of RB syndrome in dairy cows (5).

Follicular fluid (FF) provides an ideal microenvironment for the growth and development of the ovulatory follicle and the oocyte. Poor FF quality in ovulatory follicles has been frequently linked to low pregnancy rates in lactating cows. Metabolic dysfunctions and uterine infections are two main causes of low FF quality in ovulatory follicles (6–8). Furthermore, it was observed that the addition of ovulatory FF of RB Holstein heifers to oocyte maturation media disturbs oocyte maturation (9). The examination of the pre-ovulatory FF proteome showed differences in protein contents of less fertile cows, compared to those of more fertile ones (10). In addition, another study demonstrated that changes in FF composition in cows with liver problems lead to an impairment in the nuclear maturation of oocytes and the development of blastocyst (11).

Communication between the oocyte and its surrounding cumulus cells is an important event for development of a competent oocyte (12). Growth differentiation factor (*GDF9*) and steroidogenic acute regulatory (*StAR*) proteins play a major role in oocyte developmental competence (13, 14). In addition, the expression of follicle-stimulating hormone receptor (*FSHr*) has an important role in the cumulus cells expansion and the final maturation of COCs (15). Oocyte development, follicular growth, and estrous cyclicity were adversely affected in lactating cows with infections of the uterus or mammary gland (16, 17). Furthermore, it was reported that even though the clinical signs of uterine infection were disappeared, reduced fertility may persist in the infected cows for a variable length of time (18). Lipopolysaccharide (LPS) which is a major part of the outer leaflet of Gram-negative bacteria is emanated from the bacterial infections of the uterus and transported to the FF of ovulatory follicles. The higher levels of LPS in FF were associated with lower fertility in cows (19, 20). In a study, experimentally induced mastitis by Escherichia coli decreased the quality of pre-ovulatory FF, which this in turn, resulted in reduced oocyte developmental competence (21). Moreover, Shimizu et al. (22) indicated that uterine LPS may contribute to ovarian cyst development in cows. Moreover, the FF collected from pre-ovulatory follicles of cows with mastitis and endometritis was showed to contain LPS (20, 21). Very recently, it was observed that the FF LPS concentration of pre-ovulatory follicles in cows with SCE was significantly higher, compared to that of cows without SCE (23). No data is available on the effects of FF obtained from the preovulatory follicle of RB dairy cows with SCE on oocyte maturation and fertilization. With this background in mind, the current study aimed to assess the effects of FF obtained from the preovulatory follicle of RB cows with SCE on oocyte maturation, fertilization, and the expression of genes related to fertility in cultured cumulus-oocyte complexes (COCs).

## Materials and Methods

### Experiment 1

#### Animals

**Figure 1:**
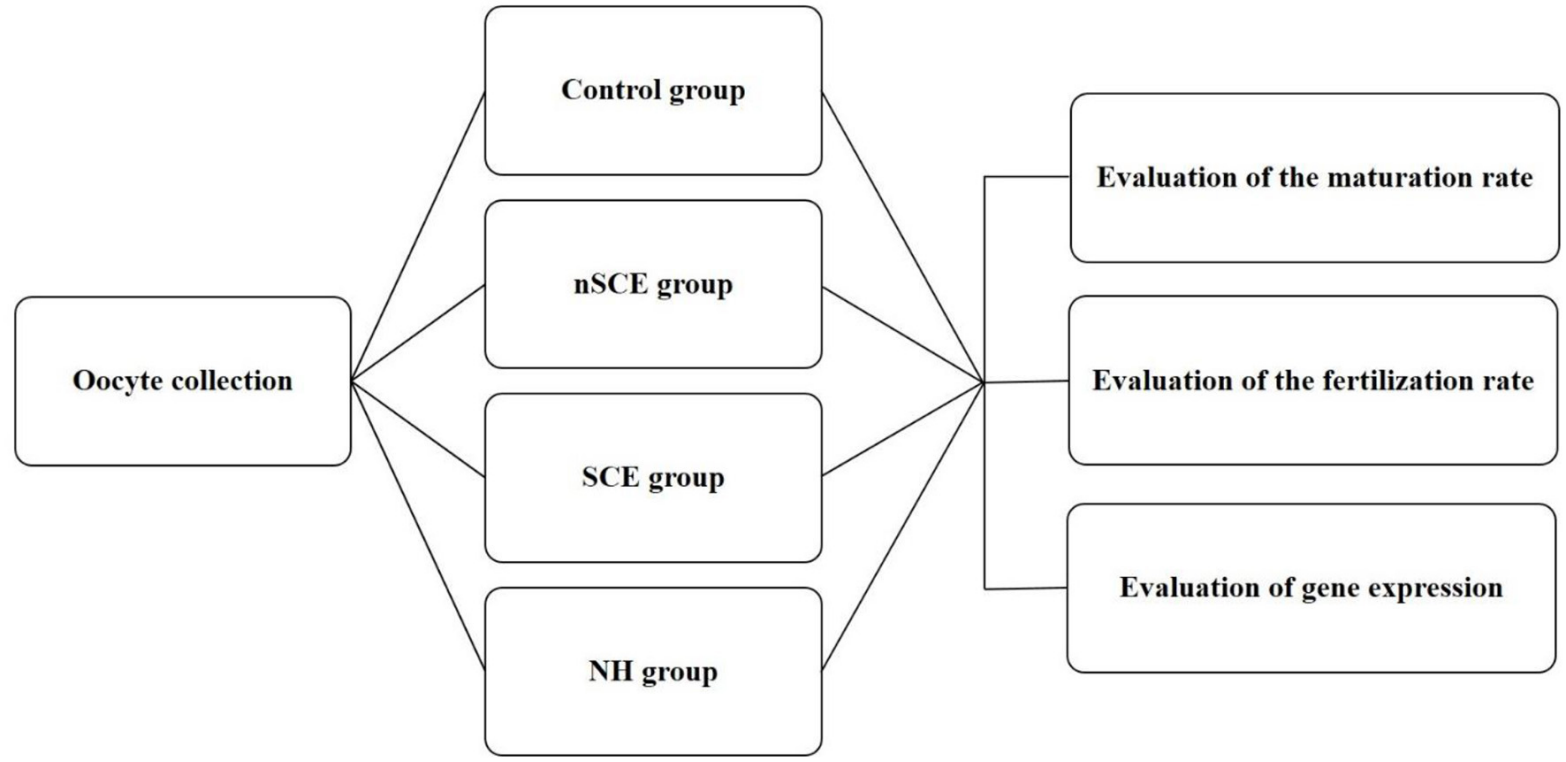
Experimental design. Control: COCs were cultured in TCM-199 medium supplemented with 10% FCS, 5 IU/mL hCG, 10 ng/ml EGF and 0.1 IU/ml human FSH for 24 h; nSCE: COCs were cultured in the TCM-199 medium supplemented with 10% filtered FF of the nSCE for 24 h; SCE: COCs were cultured in the TCM-199 medium supplemented with 10% filtered FF of the SCE for 24 h; NH: COCs were cultured in the TCM-199 medium supplemented with 10% filtered FF of the NH for 24 h.

The present study was approved by the Ethical and Research Committee of Veterinary Faculty, Shiraz University (97GCU1M1251), Iran. RB cows entered the study from a commercial Holstein dairy farm in Alborz, Iran (35° 82' N, 50° 97' E). Free-stall barns were used for the maintaining of cows and mixed feed staffs containing 40% fodder (corn silage and alfalfa) and 60% concentrated meal (soybean meal, corn, wheat bran, barley, trace minerals, and vitamins) were fed. The examination of the reproductive system of RB cows did not show any reproductive clinical signs. Means ± SD of parity, milking days, and the artificial insemination number of repeat breeder cows entered to this study were 3.3 ± 1.7, 277.9 ± 82.8, and 5.2 ± 1.6, respectively. Virgin heifers (*n* = 5) in good body condition score and 14–15 months aged were also selected as control. All heifers became pregnant in the next estrous cycle after sampling. This group of normal virgin heifers was used as the control due the presence of the maximum elements of fertility in the FF in the preovulatory follicles of these animals. The experimental design of the present study is shown in Figure 1.

#### Diagnosis of Subclinical Endometritis and Follicular Fluid Sampling

All the procedures including the diagnosis of subclinically endometriotic cows, ovulation synchronization protocol, follicular fluid sampling were conducted as described in a study by Heidari et al. (23). In brief, the estrus time of the heifers and cows were synchronized using either a two injection of PGF2α (500 μg Cloprostenol sodium, Estroplan Parnel Living Science) with 11 days interval in heifers or an Ovsynch program in RB cows. Using a cytobrush (Heinz Herenz, Hamburg, Germany), uterine secretions were collected from RB cows. For cytology, the uterine samples were spread on a slide, immersed 1 min in 90% alcohol for fixation and stained with Giemsa. RB cows with more than cut-off point of ≥3% PMNs were categorized into RBs with SCE (*n* = 10) (2, 24) and those with <3% PMNs were considered as RBs without subclinical endometritis (nSCE, *n* = 13). Six to twelve hours after detecting standing estrus in synchronized animals, transrectal ultrasonography was performed to confirm the presence of the preovulatory follicle 14–17 mm in diameter (SIUI CTS-900 Ultrasound, Shantou Institute of Ultrasonic Instruments Co., Ltd., China). The FF was aspirated as described previously by Kafi et al. (9). Briefly FF was aspirated trans-rectally using a long fine-needle covered by a hard plastic tube under the caudal epidural anesthesia. Uterine cytology showed out of 23 RB cows, 10 (43.5%) cases exhibited cytological signs of SCE whereas 13 (56.5%) cows did not.

#### LPS Assay of FF

The amount of LPS in the FF of pre-ovulatory follicles of each group was measured using an ELISA Kit (Hangzhou East biopharm CO., LTD, China). The intra-assay coefficient of variation (intra assay CV) and the inter-assay CV was obtained at <10 and <12%, respectively. Assay range and assay sensitivity were reported as 10-3000 EU/ml and 3.8 EU/ml, respectively. The Follicular fluid used in the present study was basically the same as what we used in our previous study (23). The LPS concentration in pooled FF of the preovulatory follicles of animals in NH, nSCE, and SCE groups was 410, 862, and 1063 EU/ml, respectively.

#### *In vitro* Maturation of Oocytes

The FF collected from five RB cows with a greater percentage of PMNs (SCE; PMNs ≥ 3%, *n* = 5), and the FF from five RB cows with a less percentage of PMNs (nSCE; PMNs <3%, *n* = 5) and, in addition, the FF of five normal heifers (NH, *n* = 5) were pooled separately to be substitute with serum supplement in the oocyte maturation media. Fetal calf serum (FCS) was used for serum supplement in the control group of *in vitro* maturation in Experiment 1.

For oocyte collection, the ovaries of slaughtered cows were collected and transferred to the laboratory within 2 h at 32–35°C in phosphate-buffered saline (PBS). The ovaries were washed in sterile PBS (37°C) for three times; thereafter, the cumulus-oocyte complexes (COCs) were aspirated with a 20 G hypodermic needle attached to a 10 ml disposable syringe. COCs with more than three layers of cumulus cells and a finely granulated homogenous ooplasm were selected and entered the *in vitro* maturation (25). Medium HEPES- buffered TCM-199 (Sigma, USA) supplemented with 10% FCS was used for washings of the selected COCs. In total, 735 good quality COCs were assigned to four groups. In technical control group, COCs (*n* = 204) were cultured in TCM-199 medium supplemented with 10% FCS, 5 IU/mL hCG (Karma, Germany), 10 ng/ml EGF (Sigma, USA), and 0.1 IU/ml human FSH (Follitrope, South Korea). On the other hand, in the NH group, COCs (*n* = 141) were cultured in the TCM-199 medium supplemented with 10% filtered FF of the normal heifer. In addition, in the nSCE group, COCs (*n* = 217) were cultured in TCM-199 medium supplemented with 10% filtered FF of nSCE cows. Also, in the SCE group, COCs (*n* = 173) were matured in the TCM-199 medium supplemented with 10% filtered FF of SCE cows. All cultures were carried out in four well-culture dishes (NuncTM, Denmark). In all maturation media, 50 μg/ml Gentamicin (Sigma, USA) was also added. Groups of 30–50 COCs were cultured for 24 h in a 500 μl culture media at 38.5°C in 5% CO_2_.

### Evaluation of Nuclear Maturation of Oocytes

After 24 h, the COCs were evaluated for nuclear maturation using the aceto-orcein staining method. Cumulus expansion degree was also graded using a stereo microscope from 0 (no expansion), 1 (partial expansion) to 2 (complete expansion). Also, for the evaluation of nuclear maturation of the oocytes, matured COCs were pipetted frequently to denude the oocytes which were then fixed using acetic alcohol under coverslips for 24 h. The oocytes were then stained using 1% aceto-orcein. Oocytes which did not reach to the metaphase were classified as “immature,” oocytes that chromosomes arranged in metaphase plate and peripherally located in the ooplasm were classified as ‘metaphase I (MI), oocytes with a polar body and the metaphase II plate were classified as “mature” and oocytes showed chromosomal abnormality categorized at “abnormals.”

### RNA Extraction

RNA was extracted from 50 COCs of each group using the RNeasy micro RNA extraction kit (Qiagen, Germany) as instructed by the manufacturer. In each group, four independent replicates were performed for extraction. Then, extracted RNA of all samples was quantified by Nanodrop spectrometer in order to adjust the amount of starting material in the next step.

### Real-Time Polymerase Chain Reaction

After oocyte maturation in 24 h, the expression levels of *GDF9, StAR*, and *FSHr* in COCs of NH, SCE, and nSCE groups were determined using real-time RT-PCR. For relative quantification, *GAPDH* housekeeping gene was used as the normalizer gene, and the NH group was assumed as the calibrator group.

### cDNA Synthesis

Five hundred nanogram of each extracted RNA was entered to cDNA synthesis. REVERT-L RT kit (AmpliSens biotechnologies, Korea) was used to synthesize cDNA from extracted RNA according to the manufacturer's instruction. One No-RT control for each group was included in cDNA synthesis by the omitting of reverse transcriptase enzyme from cDNA synthesis. No-RT control is a control for DNA digestion in RNA extraction step and it must be negative in the Real-time PCR test to ensure that positive results were not due to genomic DNA remnants.

### Real Time PCR

Firstly, the efficacy of each real-time PCR test was assessed by running the test on a cDNA serial dilution. If a real-time PCR had an efficiency lower than 95% or higher than 105%, the test was optimized to improve its efficiency. Power SYBR Green PCR Master Mix (Thermo Fisher Scientific, US) was consumed for the real-time PCR tests using a Corbett 6000 instrument (Qiagen, Germany). Twenty microliter of the total volume of each PCR master mix consisted of 10 μl of Power master mix, 1 μl of each primer (5 μM), 1 μl of cDNA, and 7 μl of Distilled water. The initial denaturation step was performed at the beginning of real-time PCR for 10 min at 95°C, followed by 40 cycles of 15 s at 95°C, 30 s at annealing temperature (Table 1), and 30 s at 72°C. The results were analyzed using the ΔΔCt method and the expression level of each gene in different groups was calculated in relation to the calibrator group (normal heifer).

**Table 1:** Primers used for real time RT PCR of different genes.

**Gene**	**Primer sequences (5^**′**^ to 3^**′**^)**	**Annealing temperature (^**°**^C)**	**Accession number**
*GDF9*	For: TGAAGATATGATAGCCACTAAG Rev: CTCCTCCTTACACAACAC	58	NM_174681.2
*FSHr*	For: GAATGATGTCTTGGAAGTGATAG Rev: CGATGTATAGCAGGTTGTTG	58	NM_174061.1
*StAR*	For: TGCCGAAGACCATCATCAAC Rev: GAGCCCTCAAACCCATTCAG	62	NC_037330.1
*GAPDH*	For: ATCTCGCTCCTGGAAGATG Rev: TCGGAGTGAACGGATTCG	60	NM_001034034

*Primer sequences, annealing temperatures, and accession numbers. All primers designed in this study using Beacon Designer (Primier Biosoft) software*.

### Experiment 2

#### *In vitro* Fertilization

*In vitro* oocyte maturation was carried out in four groups as described in Experiment 1. After *in vitro* maturation of the oocytes, groups of 35–50 COCs were transferred into four well-culture dishes containing 500 μl Tyrode's medium as fertilization medium. Frozen semen which previously tested in the laboratory for IVF was used for fertilization. Motile spermatozoa from frozen/thawed semen were obtained using the swim-up method and were then added to wells containing oocytes at a final concentration of 10^6^ spermatozoa ml−1 (12). Oocytes and spermatozoa were co-incubated at 38.5°C for 18 h in 5% CO_2_.

#### Assessment of the Fertilization Rate

After the fertilization period, surrounding cumulus cells of presumptive zygotes were removed by repeated pipetting which was mounted on glass slides under coverslips fixed in an acetic alcohol solution for at least 24 h and then were stained using 1% aceto-orcein. Presumptive zygotes with two pronuclei were classified as “fertilized,” while the presumptive zygotes without pronuclear formation, absent of sperm in the ooplasm, and without second polar body were classified as “non-fertilized,” presumptive zygotes with more than two pronuclei were classified as “polyspermy,” and oocytes with other nuclear structures were classified as “abnormal fertilization.”

### Statistical Analysis

The obtained data were analyzed in SPSS software (version 23). The Shapiro-Wilk test was used to assess the normality of the distribution of data sets. Differences in the cumulus expansion, maturation and fertilization rates among groups were statistically analyzed using an ANOVA test (*Post Hoc* LSD). Moreover, One Way ANOVA was applied for the statistical analysis of Real-time RT-PCR results. A *p* < 0.05 was considered statistically significant. Experiments 1 and 2 were performed in five independent replicates.

## Results

### Experiment 1

#### Percentage of Cumulus Expansion and Oocyte Nuclear Maturation

The mean (±SD) percentage of COCs demonstrated that fully expanded cumulus cells were significantly lower in SCE group than the control, nSCE and NH groups (30.4 ± 8.3 vs. 65.7 ± 12.1, 46.0 ± 12.9, and 48.8 ± 11.9, respectively; *P* < 0.05). Furthermore, the percentage of fully expanded cumulus cells between nSCE and NH was not significantly different (*P* > 0.05). The mean (±SD) percentage of nuclear maturation (M II stage) of the SCE, nSCE, NH, and control groups is shown in Table 2. The percentage of oocyte nuclear maturation in the control group was significantly higher compared to that in other groups (*P* < 0.05). Further, the mean (±SD) percentage of nuclear maturation in the nSCE group was higher than that of the SCE group (69.4 ± 10.6 vs. 61.1 ± 8.0; *P* = 0.05). Also, the percentage of oocyte nuclear maturation in the NH group was significantly higher than that in the SCE group (72.9 ± 4.9 vs. 61.1 ± 8.03; *P* < 0.05).

**Table 2:** Mean (±SD) percentages of normally mature oocytes following addition of follicular fluid collected from cows with no subclinical endometritis (nSCE), subclinical endometritis (SCE) and normal heifer (NH) to the maturation media.

**Group name**	**Number**	**MII oocytes, *n* (%)**	**Immature oocytes, *n* (%)**	**MI oocytes, *n* (%)**	**Degraded oocytes, *n* (%)**
Control	204	177 (86.7 ± 1.9)^a^	8 (3.7 ± 3.7)	15 (7.8 ± 3.3)^a^	4 (1.7 ± 2.9)
nSCE	217	149 (69.4 ± 10.6)^b^	24 (9.5 ± 12.8)	41 (19.6 ± 7.3)^b^	3 (1.3 ± 2.4)
SCE	173	105 (61.1 ± 8.0)^c^	24 (12.7 ± 16.8)	39 (22.9 ± 13.8)^b^	5 (3.1 ± 5.0)
NH	141	103 (72.9 ± 4.9)^bd^	2 (1.06 ± 2.6)	33 (24.2 ± 6)^b^	3 (1.7 ± 2.8)

*In control group, oocytes are cultured with FCS*.

*Different letters within a column showed statistically significant difference (P ≤ 0.05)*.

*MII (metaphase II), MI (metaphase I)*.

#### Real Time RT-PCR

**Figure 2:**
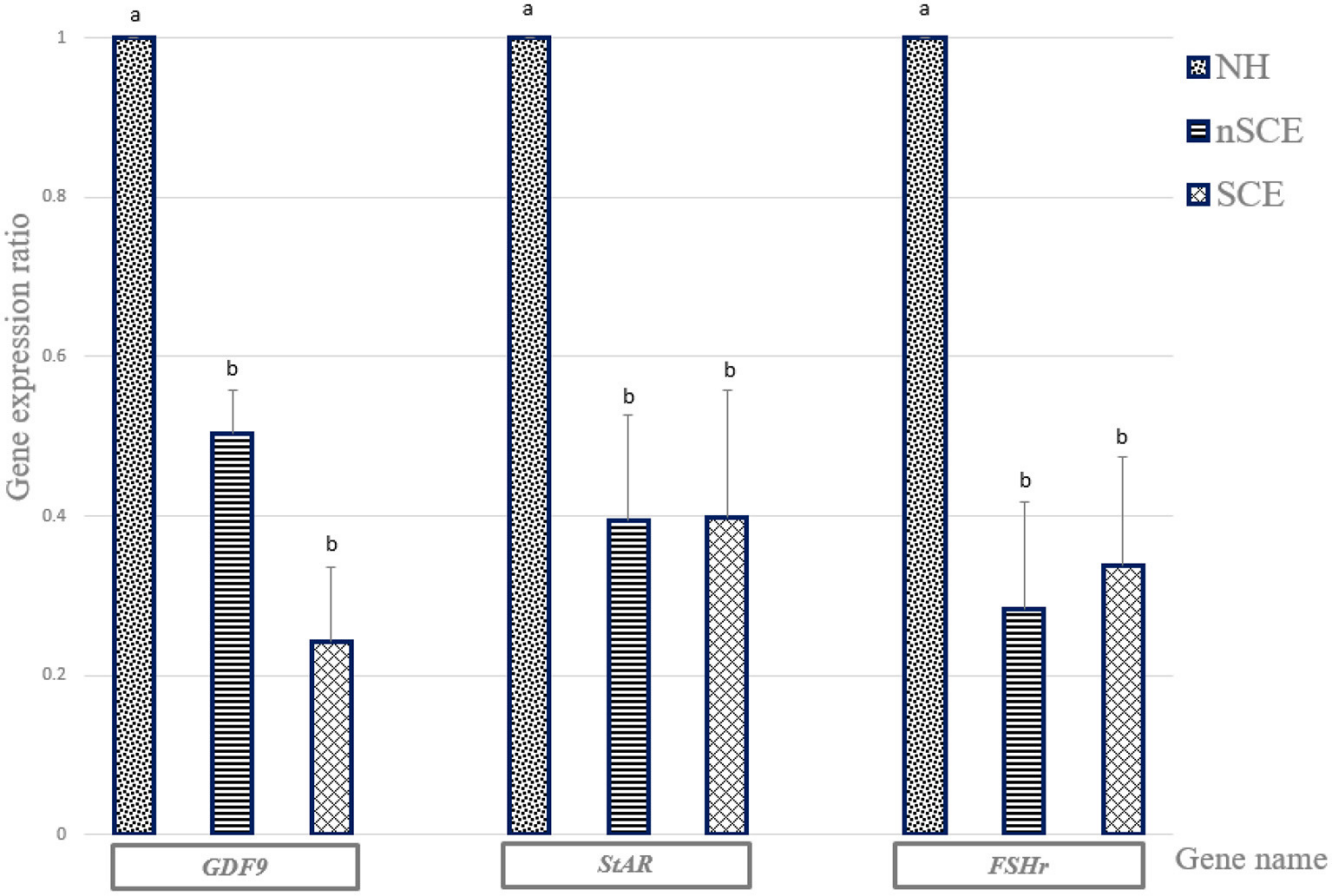
The expression ratio of *GDF9, StAR*, and *FSHr* genes. Bar charts (with standard errors) shows the expression ratio of genes in COCs cultured in the media containing pre-ovulatory FF of normal heifer (NH) and cows with subclinical endometritis (SCE), and without subclinical endometritis (nSCE). In each gene, different letters indicates statistically significant difference (*P* < 0.05).

The mRNA expression of *GDF9, StAR*, and *FSHr* in the NH group was significantly higher than those of SCE and nSCE groups (*P* < 0.05). The mRNA expressions of *GDF9, StAR*, and *FSHr* in nSCE in comparison to those of SCE were not significantly different (*P* > 0.05). The results in detail are presented in Figure 2.

### Experiment 2

The results in detail are presented in Table 3. The mean (±SD) percentage of normal oocyte fertilization was higher in the nSCE than the SCE group (58.0 ± 9.8 vs. 40.2 ± 4.5; *P* < 0.05). Furthermore, the mean (±SD) percentage of normal oocyte fertilization in the nSCE group, in comparison with the NH group was not significant (58.0 ± 9.8 vs. 48.1 ± 10.7; *P* > 0.05).

**Table 3:** Mean (±SD) percentages of normally fertilized oocytes following addition of follicular fluid collected from cows with no subclinical endometritis (nSCE), subclinical endometritis (SCE) and normal heifer (NH) to the maturation media.

**Group name**	**Number**	**Fertilization, *n* (%)**	**Non fertilized, *n* (%)**	**Polyspermy, *n* (%)**	**Abnormal fertilization, *n* (%)**
Control	132	85 (66.1 ± 12.8)^a^	39 (26.5 ± 17.5)^a^	3 (2.6 ± 2.8)	5 (4.5 ± 5.5)
nSCE	161	94 (58.0 ± 9.8)^ab^	63 (39.6 ± 8.1)^ab^	10 (0.5 ± 1.2)	3 (1.7 ± 3.2)
SCE	173	70 (40.2 ± 4.5)^c^	100 (57.8 ± 5.5)^c^	-	3 (1.8 ± 2.1)
NH	120	58 (48.1 ± 10.7)^bc^	54 (44.9 ± 9.8)^bc^	1 (0.8 ± 1.9)	7 (5.9 ± 6.9)

*Different letters within a column showed statistically significant difference (P < 0.05)*.

## Discussion

The present study strived to apply an *in vitro* model that could simulate an intra-follicular condition which occurs in the final stages of oocyte maturation in the pre-ovulatory follicles of RB cows and animals with the highest level of fertility, virgin heifers. Using FF as a component of oocyte maturation media has previously been used as a model to examine the oocyte competence in either mastitic or healthy cows (21). The results of experiment 1 showed that cumulus expansion and nuclear maturation of oocytes decreased when they were cultured *in vitro* using FF of pre-ovulatory follicles of RB cows with SCE, compared to nSCE and NH groups. The lower nuclear maturation rate in the SCE group could be due to the fact that the LPS level in the FF of pre-ovulatory follicles in RB cows with SCE was higher than that of the other groups in the present study. Similarly, Magata and Shimizu (26) using a dose-response approach found a deleterious effect of LPS on *in vitro* oocyte development in cattle. Zhao et al. (27) further showed that the LPS disrupted meiotic spindle structure and induced oxidative stress in bovine oocytes resulting in low nuclear maturation. The lower nuclear maturation rate in the SCE group in the present study explains the results of our previous study in which a lower blastocyst yield was observed when the bovine oocytes matured *in vitro* using FF of pre-ovulatory follicles of RB cows with SCE (23). One more significant finding in the present study (Experiment 2) was the lower percentage of normally *in vitro* fertilized oocytes observed in the SCE group compared to the nSCE group. The proper fertilization fundamentally depends on normal nuclear maturation and cumulus expansion to perform numerous functions required for fertilization support (28, 29).

In Experiment 1, the expression levels of *GDF9, StAR*, and *FSHr* mRNA in COCs were determined using RT-PCR. Recent researches have shown that *GDF9* protein plays a major role in oocyte developmental competence (13, 30), follicular growth, and luteinization in mammals (31). The higher oocyte nuclear maturation rates in the NH than the SCE group can be explained by the fact the *GDF9* expression level in the matured COCs in the NH group was significantly higher than those of the SCE groups in the present study. Although the nuclear maturation rate was higher in the nSCE group than that of the SCE group, however, no difference was observed in the expression level of *GDF9* in the matured COCs between the nSCE and SCE groups.

*StAR* mRNA expression in the COCs was significantly reduced in both nSCE and SCE groups in comparison with that of the NH group. This could be ascribed to the higher levels of LPS in the pre-ovulatory follicles of repeat breeder cows comparing to that of the virgin heifers. Steroidogenic acute regulatory protein is encoded by the *StAR* mRNA. *StAR* in COCs has been reported to be involved in the developmental competence of the oocyte and the embryo in bovine (32), and sheep (14). Magata et al. (33) found that a greater amount of LPS in the FF disrupted the function of theca and granulosa cells in the follicle leading to less steroid production. Also, SCE (34), and particularly LPS (35), can cause inflammation in bovine granulosa cells, which disrupts Steroidogenesis in follicles. Similarly, we observed a significant lower nuclear maturation rates in SCE groups than those of the NH group. The higher LPS level in the FF of the preovulatory follicles in the RB cows in the present study could be responsible for the lower expression of *StAR* mRNA in the matured COCs in the nSCE and SCE groups in the present study. Also, our result was in agreement with those of Campos et al. (36), Herzog et al. (37), Shimizu et al. (22), and Magata et al. (33). They observed that LPS reduced the *StAR* mRNA expression which could result in the reduction of steroid production. Our recent study (23) and others (20, 38) showed a lower concentration of 17-β estradiol associated with a high LPS in the FF of the pre-ovulatory follicles of SCE cows.

FSH has an important role in the cumulus cells expansion and the final maturation of COCs in cows and other mammals (15). The results of the present study showed a significant reduction in *FSHr* mRNA expression of the matured COCs in SCE group in comparison with that of the NH group. This could be explained by the presence of higher amount of LPS in FF of the pre-ovulatory follicles in SCE group. Although the LPS concentration was higher in the FF of preovulatory follicles of the RB cows with SCE than that of the nSCE cows in the present study, however, the pattern of expression of *GDF9, StAR*, and *FSHr* in the *in vitro* matured COCs did not explain the observed differences in the maturation and fertilization rates between the nSCE and SCE groups. Although it is difficult to explain this finding, however we assume that the effect on *GDF9, StAR*, and *FSHr* expression is associated with clinical endometritis rather than the sub-clinical endometritis. Alternatively, the bacterial type (G^+^ and G^−^) may have influenced the mRNA expression of our candidate genes as shown by Asaf et al. (21). It is important to note that significantly higher level of the expression of *GDF9, StAR*, and *FSHr* in the matured COCs of the NH group as compared to those of the RB groups suggest the presence of a disturbance in the normal function and expression of the genes involved in the fertility in RB cows. The cumulus cells surrounding the immature oocyte play a critical role in the development of the oocyte. Expansion of the cumulus cells is one of the first morphological predictive criteria of the successful completion of oocyte maturation (29). In addition, results of different researches have shown that gene expression patterns in the cumulus cells are an acceptable marker for oocyte quality (39, 40). Results of the present study showed that the mean percentage of fully expanded cumulus cells were significantly lower in SCE group than the other experimental groups. This can explain the lower nuclear oocyte maturation and fertilization rates in the SCE group comparing with that of the nSCE group in the preset study.

## Conclusions

In conclusion, the low oocyte maturation and fertilization rates could explain the disturbed fertility in RB cows specifically with subclinical endometritis. This implies the presence of poor quality of microenvironment for the final stages of oocyte development in the pre-ovulatory follicle of RB cows. Additionally, the lower fertility in RB cows could be ascribed to the lower oocyte developmental competence and less expression of *GDF9, StAR*, and *FSHr* in the cumulus-oocyte complexes.

## Data Availability Statement

The data that support the findings of this study are available from the corresponding author upon reasonable request.

## Ethics Statement

The animal study was reviewed and approved by Ethical and Research Committee of Veterinary Faculty, Shiraz University (97GCU1M1251).

## Author Contributions

MK designed this study and involved in the lab works and writing this paper. MG performed Real time RT-PCR and contributed to writing paper. MA performed the laboratory work and revised the manuscript. AM performed the follicular fluid collection. SA and YT, DVM students, were involved in the laboratory work.

## Conflict of Interest

The authors declare that the research was conducted in the absence of any commercial or financial relationships that could be construed as a potential conflict of interest.
